# Alcohol Modulates Frontal Cortex and BLA Network States Which Correlate with Future Voluntary Alcohol Consumption

**DOI:** 10.1523/ENEURO.0017-24.2024

**Published:** 2024-12-06

**Authors:** Alyssa DiLeo, Pantelis Antonodiou, Katrina Blandino, Eli Conlin, Laverne Melón, Jamie L. Maguire

**Affiliations:** ^1^Program in Neuroscience, Graduate School of Biomedical Sciences, Tufts University, Boston, Massachusetts 02111; ^2^Department of Neuroscience, Tufts School of Medicine, Tufts University, Boston, Massachusetts 02111; ^3^Department of Biology, Wesleyan University, Middletown, Connecticut 06459

**Keywords:** alcohol, basolateral amygdala, ethanol, GABA, oscillations, parvalbumin

## Abstract

Although most adults in the United States will drink alcohol in their life, only ∼6% will go on to develop an alcohol use disorder (AUD). While a great deal of work has furthered our understanding of the cycle of addiction, it remains unclear why certain people transition to disordered drinking. Altered activity in regions implicated in AUDs, like the basolateral amygdala (BLA), has been suggested to play a role in the pathophysiology of AUDs, but how these networks contribute to alcohol misuse remains unclear. Here we investigated how the impact of alcohol on the BLA network relates to alcohol exposure. We first examined the effect of acute ethanol administration on the BLA and frontal cortical networks and the relationship with subsequent voluntary ethanol consumption using the Intermittent Access paradigm. In addition, we recorded network activity from the BLA and frontal cortex throughout the Drinking-in-the-Dark-Multiple Scheduled Access paradigm to assess the impact of voluntary alcohol consumption on network states during binge and abstinence periods. Finally, we investigated the impact of acute withdrawal from chronic alcohol exposure on BLA and frontal cortex network states using the Chronic Intermittent Ethanol (vapor) paradigm. We demonstrate that across paradigms, ethanol alters low gamma band (40–70 Hz) oscillations and remarkably correlates with the extent of future voluntary ethanol consumption in the IA paradigm. These data suggest that BLA network states play a role in the mechanisms influencing voluntary alcohol intake.

## Significance Statement

Oscillatory states within and between the frontal cortex and basolateral amygdala (BLA) have been demonstrated to drive behavioral states involved in emotional processing, including negative valence processing. Given that negative emotional states/hyperkatifeia contribute to the cycle of alcohol use disorders, our previous work, demonstrating the ability of alcohol to modulate BLA network states (and thereby behavioral states), suggests that this mechanism may influence alcohol intake. Here we demonstrate a correlation between alcohol's ability to modulate oscillations in the BLA and future alcohol consumption. These findings suggest that individual variability in the sensitivity of the frontal cortex and BLA networks to alcohol influences future voluntary alcohol consumption and demonstrates a potential novel mechanism contributing to alcohol misuse.

## Introduction

Approximately 85% of adults in the United States will drink alcohol in their lifetime and about 6% will develop an alcohol use disorder (AUD; SAMHSA, 2018). Despite the large societal and health impacts, it is still unclear why certain people transition to disordered drinking (Koob and Volkow, 2009). The basolateral amygdala (BLA) is recognized as an integrative hub for processing emotional and rewarding stimuli, playing a role in fear, anxiety, social behaviors, reward, and motivation (Schoenbaum et al., 1999; Tye et al., 2011; Janak and Tye, 2015), and has been implicated in the pathophysiology of AUDs and anxiety disorders (Silberman et al., 2009; Tye et al., 2011; Agoglia and Herman, 2018). Increased activity in the BLA has been established as a hallmark feature of mood disorders, especially in those that are highly comorbid with AUDs, such as anxiety (Etkin and Wager, 2007; Zhou et al., 2010; Rau et al., 2015). Additionally, the BLA is important for reward, salience, motivation, and drug-associated cues, which play a role in relapse and the cycle of AUDs (Chaudhri et al., 2013; Sciascia et al., 2015; Wassum and Izquierdo, 2015). Clearly, BLA activity lies at the crossroads of AUD and mood disorders, highlighting our interest in the area.

Disturbed network communication in neuropsychiatric disorders is emerging as a theory for behavioral symptom manifestation (Herrmann and Demiralp, 2005; Uhlhaas and Singer, 2012; Voytek and Knight, 2015). Network oscillations are a means of communication within the brain and are emerging as critical mediators of behavior, highlighting the importance of understanding the neural mechanisms of oscillatory communication (Buzsáki and Draguhn, 2004; Gonzalez-Burgos et al., 2015; Fitzgerald and Watson, 2018; Antonoudiou et al., 2022). These network oscillations are divided into different frequency bands that often reflect different computational functions and behavioral states (Buzsáki and Draguhn, 2004; Likhtik et al., 2013; Stujenske et al., 2014; Davis et al., 2017; Ozawa et al., 2020; Antonoudiou et al., 2022; Amaya et al., 2024) and are being pursued as a potential predictive biomarker to assist in diagnosis and treatment (Javitt et al., 2008; Wu et al., 2020; Zhdanov et al., 2020). Relevant to alcohol use, EEG studies in human drinkers have found distinct differences in network activity between nondrinkers and drinkers (Rangaswamy et al., 2003; Courtney and Polich, 2010; López-Caneda et al., 2017). Additionally, previous work from our lab and others have demonstrated that specific oscillation frequencies in the BLA and prefrontal cortex orchestrate fear and safety states and are impacted by chronic stress and pharmacological treatments (Likhtik et al., 2013; Stujenske et al., 2014; Davis et al., 2017; Ozawa et al., 2020; Antonoudiou et al., 2022). Given that the BLA and prefrontal cortex play a critical role in mediating fear and anxiety (Janak and Tye, 2015), as well as alcohol consumption (Abernathy et al., 2010; Sciascia et al., 2015; Moaddab et al., 2017; Linsenbardt et al., 2019), we hypothesize that alterations in its network activity may influence drinking behavior. Preclinically, recordings of neuronal activity have been used to predict alcohol consumption (Siciliano et al., 2019; Hernandez and Moorman, 2020). However, how network states contribute to AUDs remains unclear, and understanding their role may further uncover the underlying pathophysiology of disease as well as identify potential biomarkers for risk. Thus, understanding how alcohol affects oscillations in the frontal cortex and BLA and influences alcohol consumption will be informative and may identify potential biomarkers.

Our previous work demonstrated that alcohol is capable of modulating BLA network states in response to acute alcohol exposure (DiLeo et al., 2022). Here we demonstrate that the response of the BLA network states to the first exposure to alcohol correlates with their future level of voluntary alcohol intake. We also demonstrate the ability of alcohol to modulate frontal cortex and BLA network states during voluntary consumption and altered network states associated with acute withdrawal from chronic alcohol exposure. These data suggest that there are inherent differences in the frontal cortex and BLA network which underlie the extent of alcohol use.

## Materials and Methods

### Animals

Adult male and female C57BL/6J mice, aged 8–12 weeks old, were purchased from The Jackson Laboratory (stock #000664) and group housed in temperature- and humidity-controlled housing rooms on a 12 h light/dark cycle (lights on at 7 A.M.) with *ad libitum* food and water. Animals were handled according to protocols and procedures approved by the Institutional Animal Care and Use Committee (IACUC). Female mice were maintained in an acyclic state without exposure to males. Mice were single housed and habituated to new cages for 24 h before the start of experiments.

### Stereotaxic surgery

All mice undergoing surgery were anesthetized with ketamine/xylazine (90 and 5–10 mg/kg, respectively, i.p.) and treated with sustained-release buprenorphine (0.5–1.0 mg/kg, s.c.). A lengthwise incision was made to expose the skull, and a unilateral craniotomy was performed to lower a depth electrode (PFA-coated stainless steel wire, A-M Systems) into the BLA (AP −1.50 mm, ML 3.30 mm, DV −5 mm), affixed to a head mount (Pinnacle #8201) with stainless steel screws as ground, reference, and frontal cortex electroencephalographic (EEG; AP +0.75 mm, ML ±0.3 mm, DV −2.1 mm) electrodes. Electromyographic (EMG) wires were positioned in the neck muscles.

### Acute alcohol exposure

Before starting the experimental paradigm, mice were habituated to new cages with *ad libitum* food and water for 24 h. All injections were performed 2–3 h into the light cycle (on at 7 A.M.) at the same time each day across all cohorts. The acute exposure consisted of a baseline period followed by a saline injection (0.9% NaCl, i.p.) and a subsequent 1 g/kg ethanol (EtOH) injection (20% v/v, i.p.) as previously described (DiLeo et al., 2022). Each recording period was a minimum of 60 min.

### Intermittent Access

Adult male and female C57BL/6J mice underwent an intermittent access two bottle choice (IA2BC) paradigm for 3 weeks. On Mondays, Wednesdays, and Fridays, mice received a bottle of alcohol and water for 24 h in their home cage 3 h into their light cycle and removed at the same time the following day. Bottles were measured to the nearest 10th of a gram each day. To control for evaporation and spillage, drip bottles were designated in empty cages, and the average loss per week was subtracted from individual measurements. During the first week, mice were exposed to increasing concentrations of alcohol (w/v) starting with 3, 6, and 10% before being introduced to 20% alcohol for the rest of the paradigm. Fluid intake was measured in milliliters to calculate the gram per kilogram intake and preference (%). Control groups that received only water bottles were run concurrently with the alcohol-exposed groups. Bottle position on cages was switched each session and counterbalanced throughout the groups to ensure place preference was not established. Mice were split into high or low drinking groups based on a mean split of the total average drinking across all 3 weeks of the IA paradigm. Those above the mean were designated as high drinkers, and those below the mean were designated as low drinkers.

### Drinking-in-the-Dark-Multiple Scheduled Access paradigm

The Drinking-in-the-Dark-Multiple Scheduled Access (DID-MSA) procedure was performed as previously described (Melón et al., 2018) and adapted from Bell et al. (2011) and Melón et al. (2013). Briefly, beginning at lights out, mice are given access to water or a 20% unsweetened alcohol solution (95% ethanol, Pharmco Products) during three, 1 h drinking sessions, each separated by 2 h breaks where only water was provided. Volumes consumed were recorded for each 1 h binge-drinking session and summed for the day.

### Chronic Intermittent Ethanol paradigm

The Chronic Intermittent Ethanol (CIE) paradigm was performed in vapor chambers acquired from La Jolla Alcohol Research Institute. Animals were randomly assigned to either vapor (CIE) or control (air) conditions. The target blood ethanol concentration (BEC) range for the CIE group was 180–290 mg/dl. For 2 weeks Monday through Thursday, animals were placed in chambers for 16 h (5:00 P.M. to 9:00 A.M). Animals were weighed the first day of each week. All animals received pyrazole (1 mmol/kg) via intraperitoneal (i.p.) injections, and CIE mice received a loading dose of ethanol (1.6 g/kg, i.p.). All animals received 0.3 ml subcutaneous 0.9% saline. After removal from chambers, animals were tested on rotarod for intoxication levels and received 0.3 ml of subcutaneous saline. They recovered for 8 h and were abstinent Friday through Monday. CIE animals received hydrogel and nutrient gel and heat pad for postethanol recovery as well as during abstinence days. Submandibular blood was collected from one CIE and one air mouse on Thursday each week and plasma isolated for measurement of BEC.

### LFP recordings and spectral analysis

LFP recordings were acquired using Lab Chart software (ADInstruments). Spectral analysis of the LFP/EEG signal was performed using the short-time Fourier transform (STFT) with a 5 s Hann window and 50% overlap. The power line noise (59–61 Hz) was removed and values were filled using nearest interpolation. Outliers in each spectrogram were identified using a two-stage process. Firstly, a time series was obtained from the mean power across frequencies of each spectrogram. Large deviations, defined as those greater than the mean plus 4 times the standard deviation, were replaced with the median of the nonoutlying data. Then, a sliding window method was applied to detect more subtle outliers based on local context, using 5× the median absolute deviation (MAD) of each 5 min window. Resulting outliers were removed and replaced with forward fill interpolation of the nearest values. All resulting time series, obtained from the mean power across frequencies, were manually inspected to remove bad regions that were replaced with median values for each frequency. Any resulting power spectral densities (PSDs) with no apparent peaks were rejected and were not included for further analysis. For the DID-MSA paradigm, each PSD was normalized to its total power across all frequencies analyzed (1–120 Hz). Then a value for each frequency band was obtained across all binge and break sessions. The normalized power for each frequency band and brain region were converted to *z*-scores.

### Statistical analysis

Data were analyzed using Prism software (GraphPad) and Python. All statistical tests are reported in detail in the statistical tables as Extended Data. Briefly, for analysis of effects on LFP frequencies, repeated measures of two-way ANOVAs were performed to detect the significance of frequency, treatment, sex, or genotype. A mixed-effects model was used if values were missing across days. A post hoc Šídák's multiple-comparisons test was performed to identify significant differences in specific groups. *p* values <0.05 were considered significant (*p < 0.05, **p < 0.01, ***p < 0.001, ****p < 0.0001). All *n* values for each treatment group are also included in the figure legends. For LFP/EEG data, outliers were further removed using Tukey's fences *K* = 2.5. For the DID-MSA data, boxplots were created in PRISM, and significance was tested using two-way ANOVAs (frequency vs session) followed by multiple *t* test adjusted using the Šídák method. Scatterplots with regression line were created using the seaborn lmplot. Correlation was assessed using Pearson’s correlation, and significance was assessed using a permutation of the shuffled values (*N* = 10,000 permutations). When two or more correlations were run as part of the same group of tests, as was the case for the DID-MSA paradigm, the resulting *p* values were corrected for multiple comparisons using false discovery rate (Benjamini/Hochberg method).

## Results

### Intermittent access escalates alcohol intake in C57BL/6J mice

**Figure 1.**
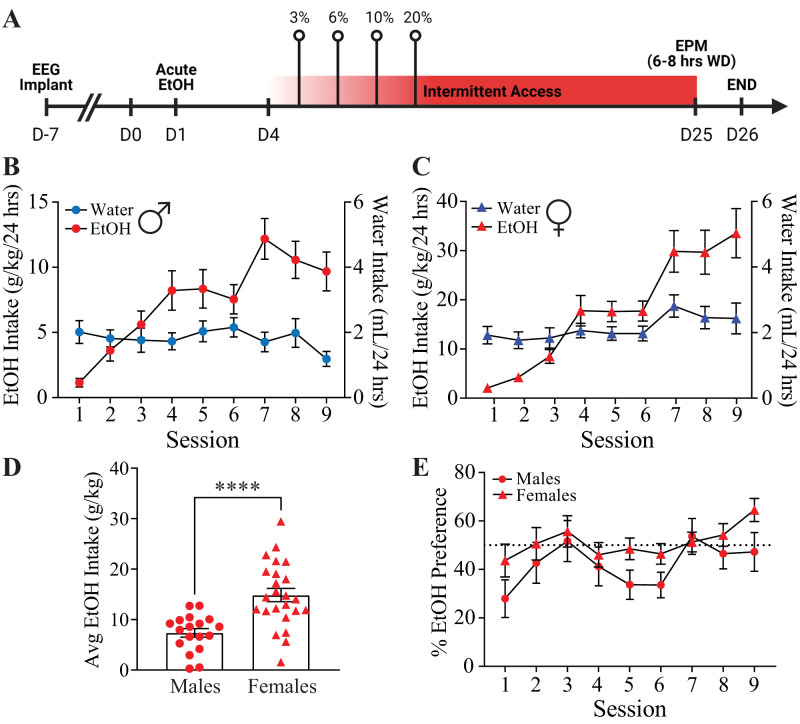
Intermittent access escalates alcohol intake in C57BL/6J mice. ***A***, Schematic of the experimental design with a timeline of LFP/EEG recording in response to acute ethanol exposure preceding the 3 week IA2BC procedure and subsequent behavioral assessments. The average water (blue) and ethanol (red) consumed per session throughout the IA2BC paradigm in males (***B***; *N* = 18) and females (***C***; *N* = 24). ***D***, The average alcohol intake consumed during the IA2BC paradigm is higher in females than in males with no difference in alcohol preference (***E***). Individual and mean ± SEM values are graphed. ****p < 0.0001.

Since we determined that acute alcohol could modulate BLA network states in C57BL/6J mice (DiLeo et al., 2022) and we have shown that disturbed network states can influence mood disorders and behavior (Antonoudiou et al., 2022; Walton et al., 2022), we hypothesized that the BLA network response to acute alcohol may predict or correlate with voluntary alcohol intake. To test this hypothesis, we recorded LFPs and EEG in the BLA and frontal cortex of male and female C57BL/6J mice during acute alcohol exposure (Fig. 1) and then subjected them to a 3 week IA2BC paradigm (Fig. 1*A*). IA2BC escalated alcohol intake, but not water intake, from session 1 to session 9 in males and females (Fig. 1*B*,*C*). Females (14.86 ± 1.319) had a higher average alcohol intake over 3 weeks compared with males (7.37 ± 0.854; Fig. 1*D*), a finding which is well established for this and other voluntary drinking paradigms (Hwa et al., 2011). Preference for alcohol did not significantly change from sessions 1 to 9, and there were no differences between males and females (Fig. 1*E*). Total fluid intake and body weights were not different between water and alcohol groups for male (Extended Data Fig. 1-1*A,B*) or for female C57BL/6J mice apart from a small but statistically significant increase in body weight (Week 1, 19.37 ± 0.35 g; Week 2, 20.05 ± 0.463 g; Week 3, 20.26 ± 0.452 g; Extended Data Fig. 1-1*C,D*).

10.1523/ENEURO.0017-24.2024.f1-1 Figure 1-1 **The IA2BC paradigm does not alter fluid intake or body weight.** Total fluid intake (A, C) and body weights (B, D) in male (A-B; water *n* = 7; EtOH *n* = 18) and female (C-D; water *n* = 12; EtOH *n* = 24) water drinkers (blue) or ethanol exposed (red). Download Figure 1-1, TIF file.

### BLA and frontal cortex response to acute alcohol correlates with voluntary drinking levels

**Figure 2.**
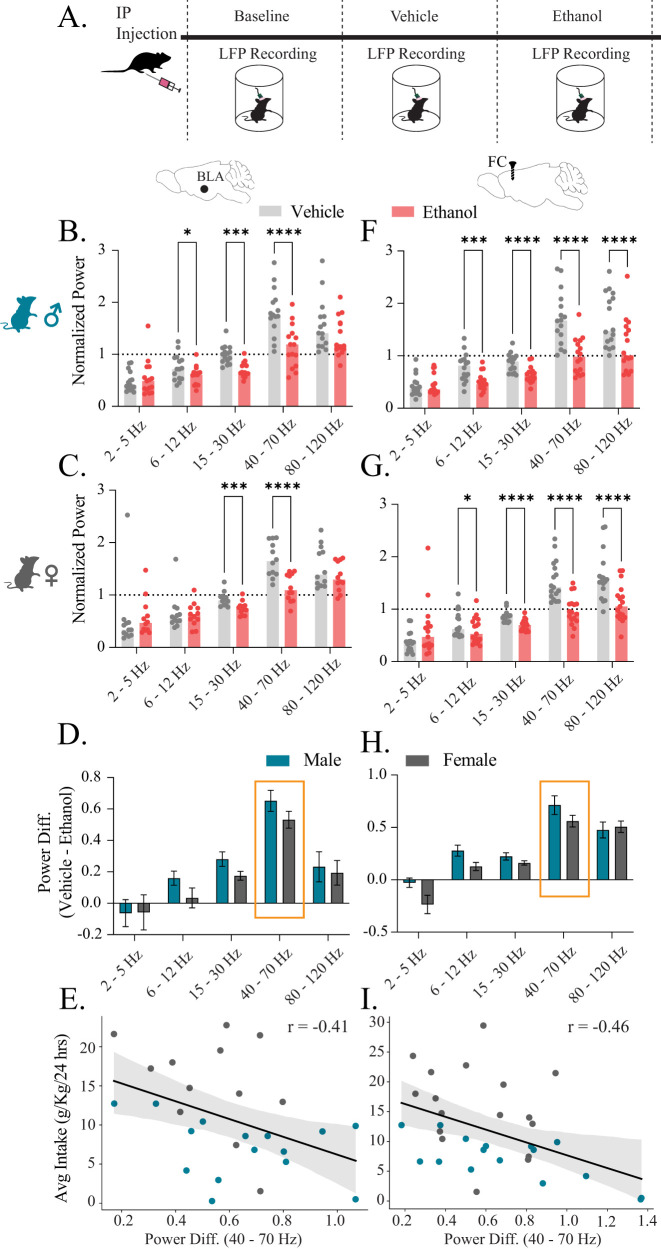
Gamma oscillations in the BLA correlate with the extent of voluntary alcohol consumption. ***A***, Experimental paradigm of acute alcohol administration and concurrent LFP/EEG recordings. BLA LFP (***B–E***), Frontal cortex EEG (***F–I***). Normalized power to baseline across frequency bands in male (***B***, ***F***) and female (***C***, ***G***) mice after vehicle or ethanol IP injections. ***D***, ***H***, Power difference (vehicle–ethanol) was not different between male and female mice. The biggest power difference for both male and female mice was for the low gamma band indicated with orange outline. ***E–I***, There is a significant negative correlation between the ability of acute alcohol to modulate gamma oscillations and the subsequent amount of voluntary alcohol consumed. Dots represent individual animals and error bars represent SEM. BLA: *N*male = 15, *N*female = 12; frontal cortex: *N*male = 16, *N*female = 17. *p < 0.05, **p < 0.01, ***p < 0.001, ****p < 0.0001.

To understand if there was a relationship between the oscillatory dynamics during acute alcohol exposure to voluntary drinking levels from the I2ABC paradigm, we first analyzed oscillatory states in response to acute alcohol exposure (Fig. 2*A*). We found that alcohol administration robustly suppressed network dynamics across multiple oscillatory bands in the BLA and frontal cortex in both male and female mice (Fig. 2*B*,*C*,*F*,*G*; Extended Data Fig. 2-1). These effects of ethanol on the network were not different between male and female mice in either BLA (Fig. 2*D*) or frontal cortex (Fig. 2*H*).

10.1523/ENEURO.0017-24.2024.f2-1 Figure 2-1 **Power spectral densities and time range selection used for analysis of Figure 2.** BLA (A) and frontal cortex (B) raw power spectral densities in log scale indicating clear peaks in the 2–5  Hz, 6–12  Hz, and 40–70  Hz frequency range (Analysis includes full 60 minutes per treatment condition (Baseline, Vehicle, Ethanol). BLA (C) and frontal cortex (D) low gamma (40–70  Hz) power across time with orange box highlighting time selected (2-7 minutes) for analysis in Figure 2. Download Figure 2-1, TIF file.

Importantly, the largest effect observed across sex and region was suppression of the low gamma band (40–70 Hz; Fig. 2*D–H*). Therefore, we compared the low gamma power difference between acute ethanol and vehicle treatments to voluntary ethanol drinking levels from the I2ABC paradigm. We found that both BLA and frontal cortex gamma power (40–70 Hz) had a significant negative correlation (BLA, *r *= −0.41; frontal cortex, *r *= −0.46) with average alcohol intake (Fig. 2*E*,*I*), demonstrating that the animals in which acute alcohol exposure least impacted network activity consumed more alcohol. Indeed, a linear regression model (*R*^2 ^= 0.472; *F*_(3,56)_ = 16.66; *p* < 0.0001) showed that both gamma power (*p* = 0.004) and sex (*p* < 0.0001) were significant contributors to the model with a dependent variable the average voluntary alcohol consumption (Extended Data Table 5). These data suggest that the combination of low gamma power in BLA/frontal cortex and sex could be powerful predictors of the extent of voluntary alcohol consumption and the anxiolytic effects of alcohol.

### Altered BLA network states translates to other models of alcohol exposure and withdrawal

**Figure 3.**
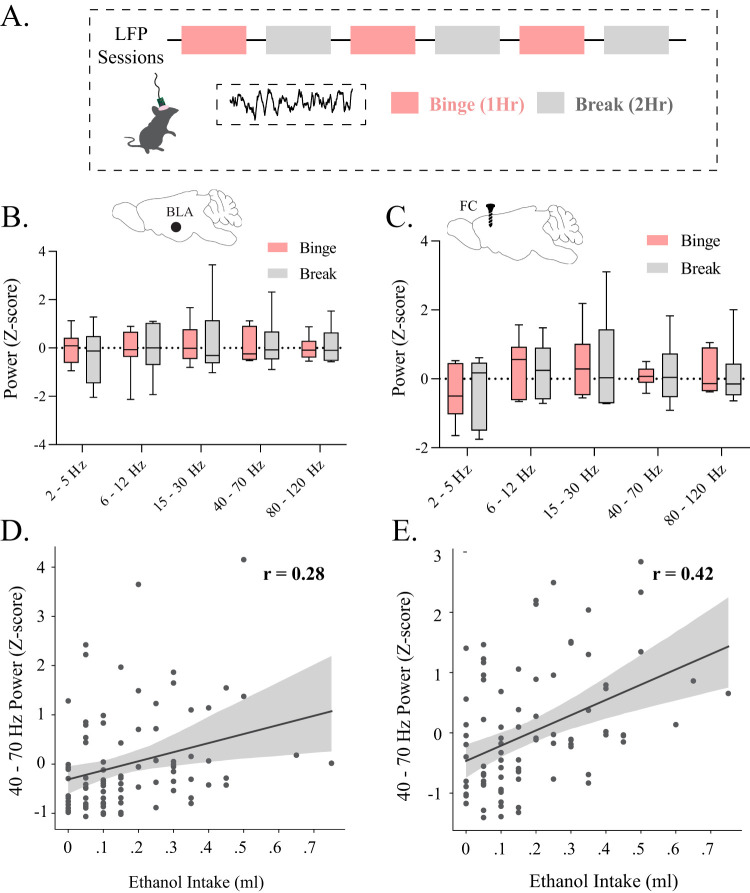
Binge drinking alters oscillatory states in the BLA. ***A***, A schematic of the DID-MSA paradigm. The average power of oscillations across frequencies in the BLA [*n* = 7 (3 male, 4 female)] and (***B***) frontal cortex [*n* = 6 (2 male, 4 female)] during binge and break periods of the DID-MSA paradigm. There is a positive correlation between oscillatory states in (***D***) the BLA [*n* = 95 sessions (11 mice)] and (***E***) frontal cortex [*n* = 89 sessions (11 mice)] between oscillations in the gamma range and the amount of ethanol consumed in the DID-MSA binge periods.

To better appreciate the impact of voluntary alcohol intake on BLA network states, we employed the DID-MSA paradigm (Fig. 3*A*). The power of oscillations across frequency bands is quantified across binge and break periods throughout the DID-MSA protocol in the BLA and frontal cortex (Extended Data Fig. 3-1). While there were no significant differences in the power of the oscillations between binge and break sessions in either the BLA (Fig. 3*B*) or frontal cortex (Fig. 3*C*), the power of oscillations within the low theta frequency (2–5 Hz) in the BLA (*r *= −0.26; Extended Data Fig. 3-2*A*, left) and frontal cortex (*r *= −0.37; Extended Data Fig. 5-2*B*, left) was inversely correlated with the extent of alcohol intake. In contrast, the power of low gamma oscillations (40–70 Hz) in both the BLA (*r *= −0.28; Fig. 3*D*) and frontal cortex (*r *= −0.42; Fig. 3*E*) was positively correlated with the amount of ethanol intake. These data demonstrate that the more alcohol consumed voluntarily the greater the modulation of BLA network activity. These findings demonstrate that alcohol influences BLA and frontal cortex network states during voluntary consumption in relation to the extent of alcohol consumed.

10.1523/ENEURO.0017-24.2024.f3-1 Figure 3-1 **Oscillatory states during binge drinking.** The average power of oscillations across frequencies in the (A) BLA (n = 7 (3 male, 4 female)), (B) frontal cortex (n = 7 (3 male, 4 female)) during individual binge and break sessions throughout the DID-MSA protocol. Download Figure 3-1, TIF file.

10.1523/ENEURO.0017-24.2024.f3-2 Figure 3-2 **Power in BLA and frontal cortex but not coherence correlates with voluntary ethanol intake.** Relationship between (A) BLA power (n = 95 sessions (11 mice)), (B) frontal cortex power (n = 89 sessions (11 mice)) with the amount of ethanol consumed in the DID-MSA binge periods. Download Figure 3-2, TIF file.

**Figure 4.**
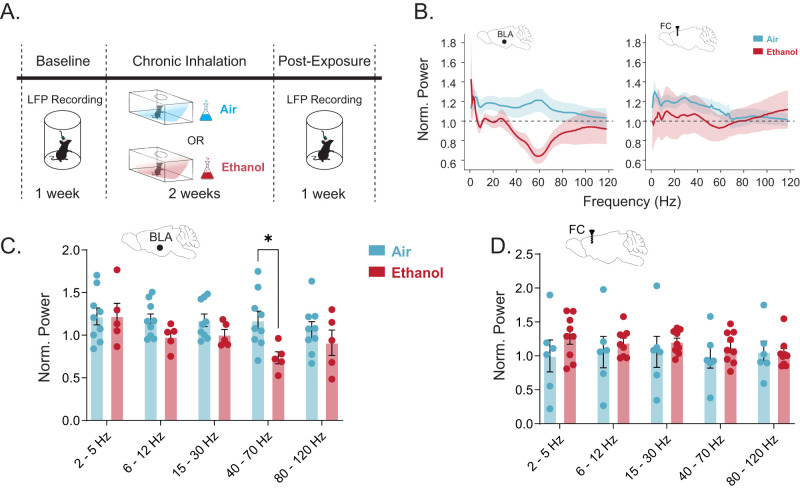
CIE reduces gamma band oscillations in the BLA. ***A***, Schematic of the CIE paradigm. LFP recordings were performed before and after chronic inhalation of air or ethanol. ***B***, Power spectral density plots normalized to baseline period in BLA (air, *n* = 9; ethanol, *n* = 5) and FC (air, *n* = 9; ethanol, *n* = 6), where shaded regions indicate SEM. Summary bar plots of normalized power in (***C***) BLA and (***D***) FC, where dots represent individual animals and error bars represent SEM. *p < 0.05.

The previous experiments examined the impact of acute alcohol administration and acute voluntary intake on BLA network states. To examine the impact of chronic alcohol exposure and withdrawal on BLA network states, we utilized the CIE paradigm (Fig. 4*A*). We found that withdrawal following chronic ethanol exposure is associated with a decrease in gamma power in the BLA (Fig. 4*B*,*C*), but not the frontal cortex (Fig. 4*B*,*D*), compared with air exposed controls. Collectively, these data demonstrate that voluntary alcohol exposure modulates BLA network activity and withdrawal is associated with opposing effects on BLA network states, which may alter the ability of alcohol to modulate BLA network states and promote increased alcohol intake.

## Discussion

It has been well documented that network communication within and between the frontal cortex and BLA is important for driving distinct behavioral outcomes (Likhtik et al., 2013; Calhoon and Tye, 2015; Janak and Tye, 2015; Tovote et al., 2015; Chen et al., 2021). In light of our previous work demonstrating that alcohol is capable of modulating BLA network states, we proposed that the ability of alcohol to modulate frontal cortex and BLA network dynamics may influence drinking behavior. In order to test the hypothesis that oscillatory states within and between the frontal cortex and BLA in response to acute alcohol could predict or correlate with future voluntary alcohol drinking, we recorded network activity in the frontal cortex and BLA during an acute alcohol exposure combined with an IA2BC paradigm for 3 weeks to monitor alcohol intake in male and female C57BL/6J mice. We observed that acute alcohol exposure robustly suppressed network oscillations with the biggest changes seen in the low gamma band (40–70 Hz) in both the BLA and frontal cortex independent of sex which is consistent with our previous results and results found by others (Henricks et al., 2019; DiLeo et al., 2022). These findings replicated previous work from our laboratory (DiLeo et al., 2022) and the increased sample size enabled us to resolve more widespread changes in oscillatory states across frequency bands in the BLA and extend these findings in the frontal cortex in response to acute alcohol exposure. While it is well established that communication between the frontal cortex and BLA influences behavioral outcomes, consistent with these current findings, we cannot rule out that alcohol may modulate other networks and circuits which also influence drinking behavior. However, these data do indicate that alcohol is capable of altering the rhythmicity and/or synchronicity of principal cell ensembles in the frontal cortex and BLA, which may be mediated by impacts on PV interneurons that are crucial contributors to oscillatory activity and generation of oscillations in the BLA (Cardin et al., 2009; Sohal et al., 2009; Antonoudiou et al., 2022). These effects of alcohol on oscillations in the BLA and frontal cortex are unique from other GABA modulators, including diazepam and allopregnanolone (Antonoudiou et al., 2022). Interestingly, we also showed that the gamma power reduction following acute ethanol administration negatively correlated with voluntary drinking of ethanol in the IA2BC paradigm indicating that the animals whose gamma network activity were least impacted consumed more alcohol. This finding reinforces the question of whether alterations of network activity following acute ethanol could be predictive of future drinking behavior in humans.

We also examined the relationship between oscillatory states in BLA and frontal cortex during voluntary alcohol consumption in the DID-MSA paradigm. We demonstrated that voluntary alcohol consumption in this paradigm was capable of altering BLA network states which were directly proportional to the extent of alcohol consumption. We also demonstrate perturbations in BLA network states following withdrawal from chronic ethanol exposure (Fig. 4), resulting in a suppression of gamma oscillations in the BLA. Given that the ability of alcohol to decrease gamma oscillations was associated with increased alcohol intake (Fig. 3), these data may indicate that disruptions in BLA network states following CIE may contribute to increased and/or escalated alcohol intake (Becker and Baros, 2006; Griffin et al., 2009a,b; Lopez et al., 2012). Further, increased voluntary alcohol intake in the DID-MSA paradigm correlates with increased gamma oscillation power (Fig. 3). Following CIE, it may require an increased amount of alcohol to modulate BLA network states, resulting in increased and/or escalated alcohol intake. Future studies are required to fully understand how these changes may relate to the cycle of AUDs. Given the role of BLA network states in emotional processing, it is also conceivable that these changes may contribute to hyperkatifeia and a drive for increased alcohol consumption.

Interestingly, we did not find sex differences in acute alcohol's effects on BLA network activity here even though male and female mice have been shown to have differing sensitivity to the rewarding and aversive properties of alcohol (Vetter-O’Hagen et al., 2009; Hwa et al., 2011; Flores-Bonilla and Richardson, 2020). Sex differences have been reported in neural oscillations in major depressive disorder and rodent models of post-traumatic stress disorder, suggesting we may see similar differences in responses to alcohol (Anker and Carroll, 2011; Fenton et al., 2016). Sex differences in alcohol-related anxiety-like behavior is also well-documented (Rhodes et al., 2005; Barkley-Levenson and Crabbe, 2015) and may be related to altered BLA network states given the relationship to behavioral states (Likhtik et al., 2013; Stujenske et al., 2014; Davis et al., 2017; Ozawa et al., 2020; Antonoudiou et al., 2022). Additionally, sex differences have been reported in neural oscillations in major depressive disorder with oscillatory signatures of susceptibility (Theriault et al., 2021).

Additional research is required to fully understand the mechanisms mediating the impact of alcohol on BLA network states. Despite these remaining questions, the current study demonstrates that the ability of alcohol to modulate BLA network states correlates with future voluntary alcohol consumption which necessitates digging deeper into the mechanisms through which alcohol impacts BLA network states and the inherent differences in the responsivity of this network to the effects of alcohol. Further, we demonstrate unique effects of alcohol withdrawal on BLA network states which we propose may contribute to negative affective states and hyperkatifeia and drive to increase alcohol intake. Future studies are required to evaluate the relevance of these BLA network states to the cycle of AUDs.

## Synthesis

Reviewing Editor: Sam Golden, The University of Washington Decisions are customarily a result of the Reviewing Editor and the peer reviewers coming together and discussing their recommendations until a consensus is reached. When revisions are invited, a fact-based synthesis statement explaining their decision and outlining what is needed to prepare a revision will be listed below. The following reviewer(s) agreed to reveal their identity: NONE.

Dear Dr. Maguire,

I am pleased to share that your revision has been reviewed by the same two experts, who feel the manuscript is now appropriate for publication. Please note that Reviewer 2 has a minor comment that can be corrected during the proofing period.

Thank you again for the revised manuscript.

Reviewer 1:

The revised manuscript is significantly improved. While I was enthusiastic about the mechanistic molecular data in previous versions, removing it provides a much more streamlined, clear story. The complete table of statistics alleviates my concerns with the remaining data in this version. All other concerns were addressed by removing several pieces of data. I commend the authors on the improved manuscript.

Reviewer 2:

The authors have addressed all of my concerns. Thanks to the streamlining of the manuscript, the clarity of the central message is much improved. With the inclusion of the stats tables, the statistical analysis of the studies can now be evaluated. The ms in its current form should be accepted for publication in eNeuro.

I would ask that the authors and editor verify their included statistics before publication, as (1) post-hoc tests on non-significant interaction effects seem to be unnecessarily included for figures 1E, 2H (M vs F within each band), and 4D; (2) Statistical information for figure 3D, E is missing. This should not necessitate another round of review.
