# Supplementary figures and images for: Alcohol Modulates Frontal Cortex and BLA Network States Which Correlate with Future Voluntary Alcohol Consumption
Source: eNeuro. 2024 Dec 6;11(12):ENEURO.0017-24.2024. doi: 10.1523/ENEURO.0017-24.2024 (PMC11649967; doi:10.1523/ENEURO.0017-24.2024)

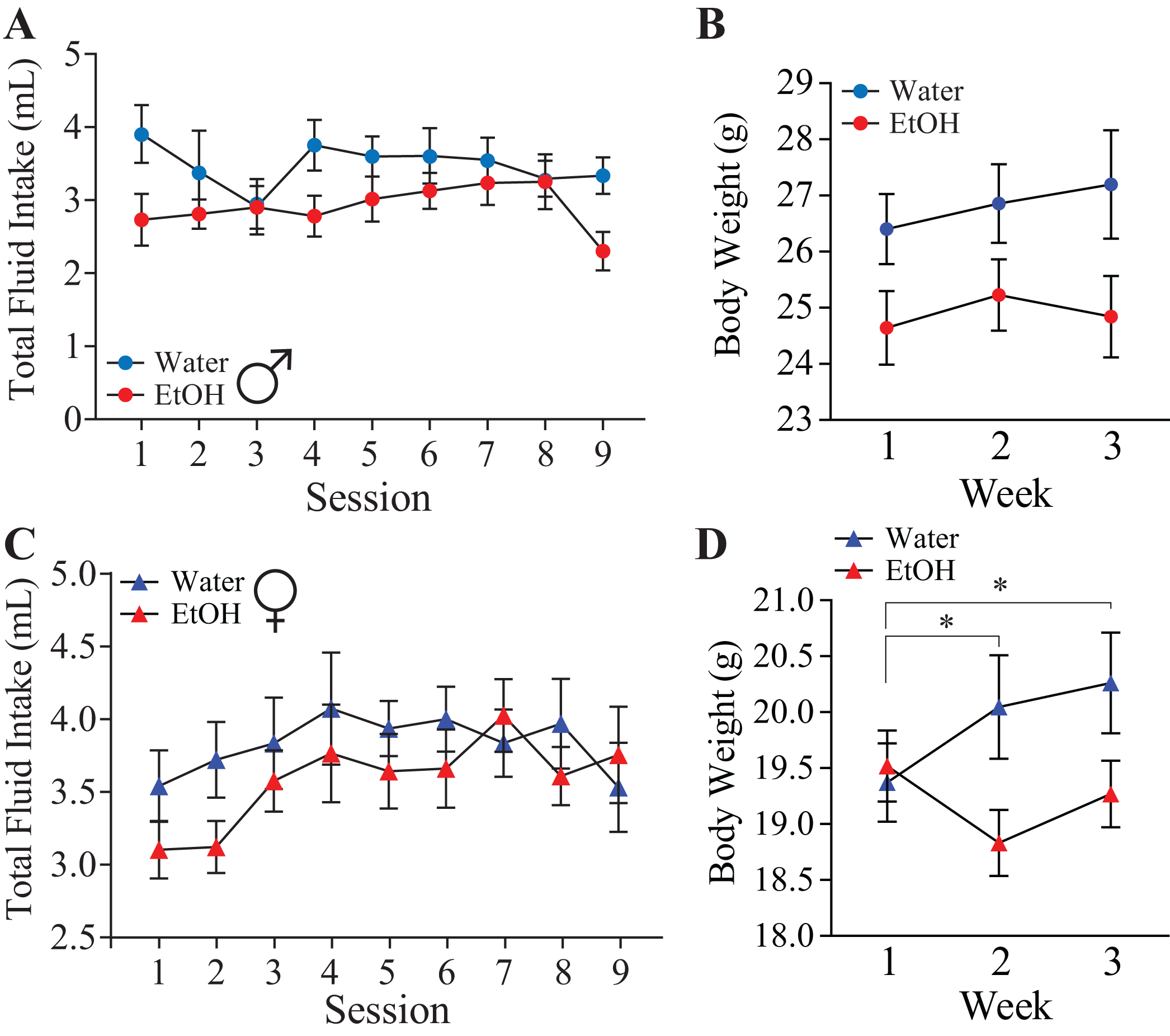

Supplement: Figure 1-1 — The IA2BC paradigm does not alter fluid intake or body weight. Total fluid intake (A, C) and body weights (B, D) in male (A-B; water n = 7; EtOH n = 18) and female (C-D; water n = 12; EtOH n = 24) water drinkers (blue) or ethanol exposed (red). Download Figure 1-1, TIF file. [file eneuro-11-ENEURO.0017-24.2024-s001.tif]

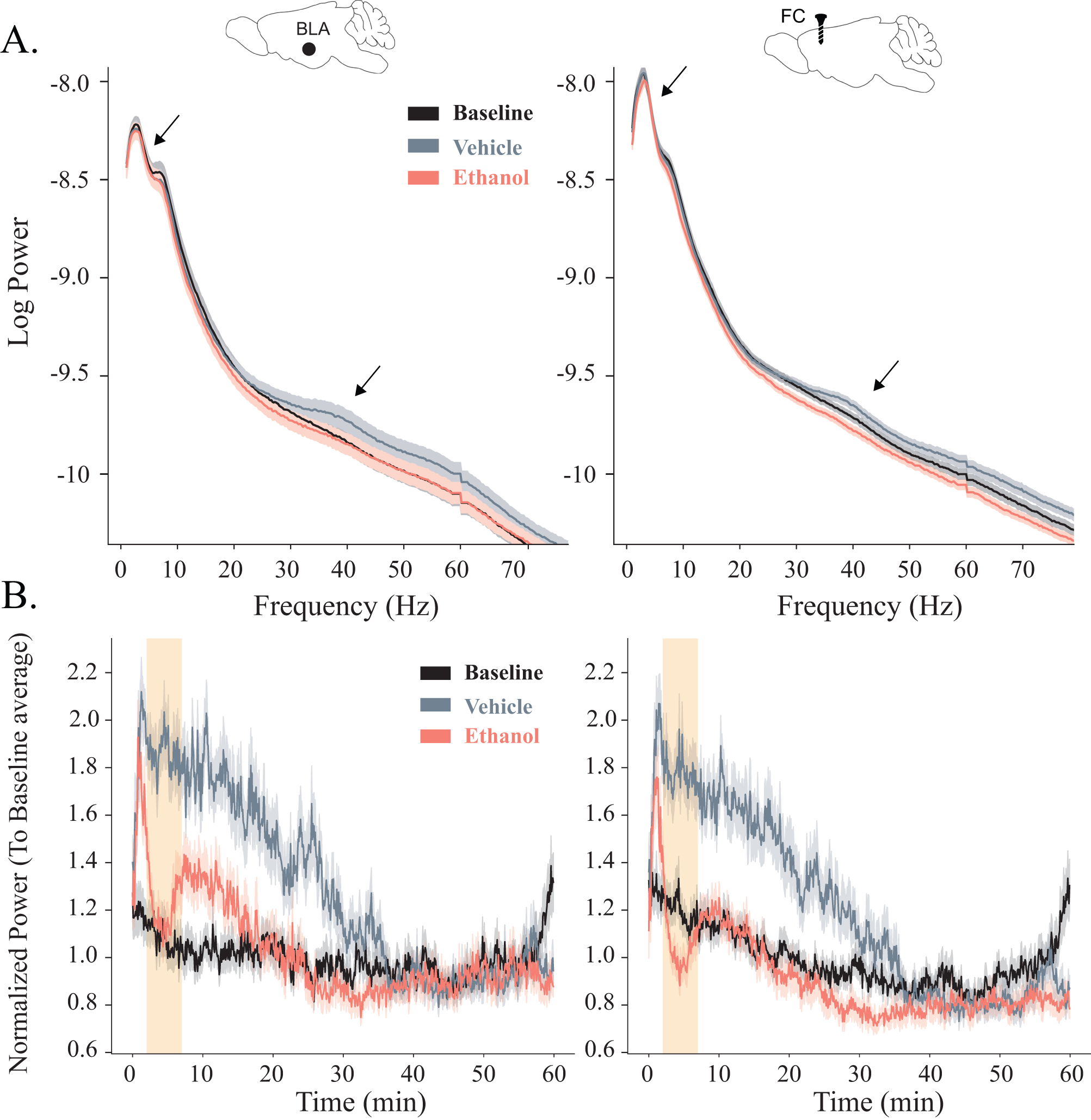

Supplement: Figure 2-1 — Power spectral densities and time range selection used for analysis of Figure 2. BLA (A) and frontal cortex (B) raw power spectral densities in log scale indicating clear peaks in the 2–5 Hz, 6–12 Hz, and 40–70 Hz frequency range (Analysis includes full 60 minutes per treatment condition (Baseline, Vehicle, Ethanol). BLA (C) and frontal cortex (D) low gamma (40–70 Hz) power across time with orange box highlighting time selected (2-7 minutes) for analysis in Figure 2. Download Figure 2-1, TIF file. [file eneuro-11-ENEURO.0017-24.2024-s002.tif]

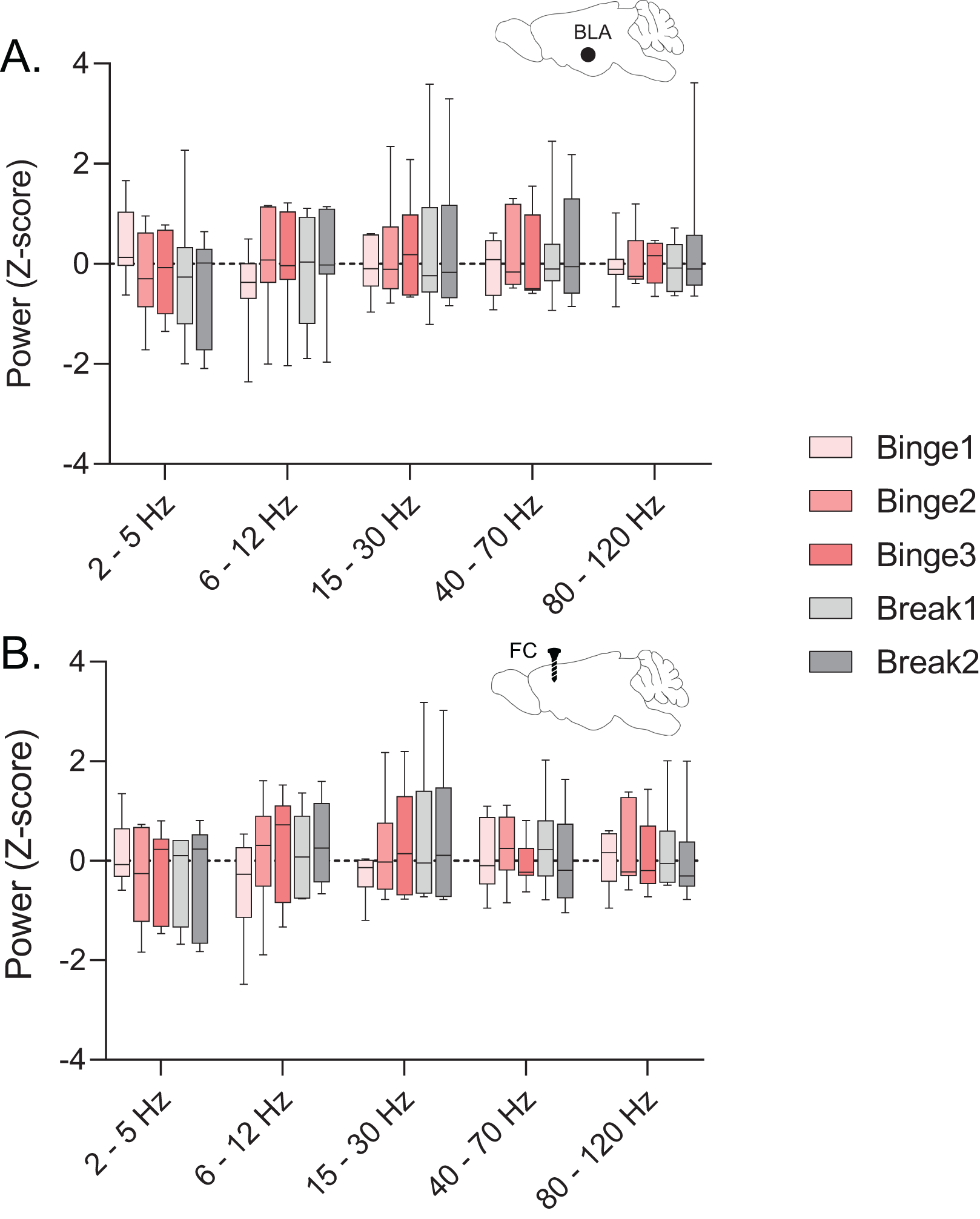

Supplement: Figure 3-1 — Oscillatory states during binge drinking. The average power of oscillations across frequencies in the (A) BLA (n = 7 (3 male, 4 female)), (B) frontal cortex (n = 7 (3 male, 4 female)) during individual binge and break sessions throughout the DID-MSA protocol. Download Figure 3-1, TIF file. [file eneuro-11-ENEURO.0017-24.2024-s003.tif]

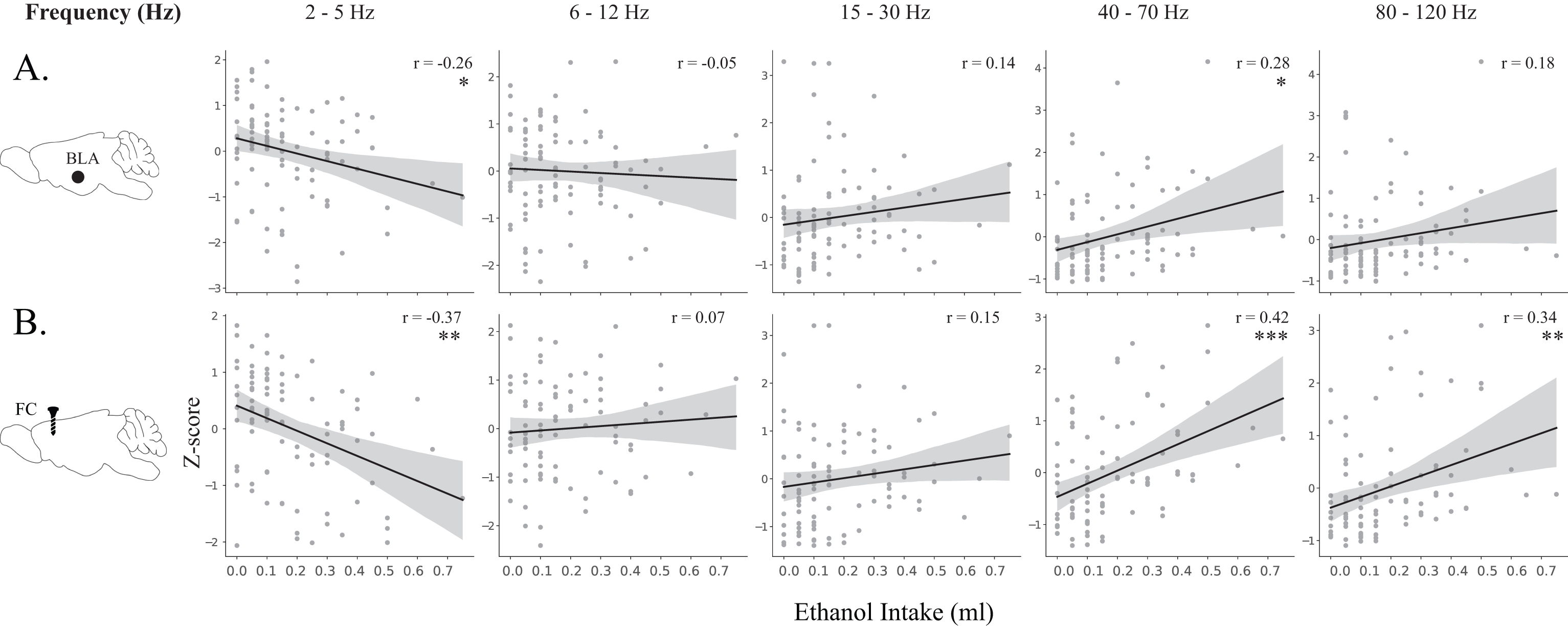

Supplement: Figure 3-2 — Power in BLA and frontal cortex but not coherence correlates with voluntary ethanol intake. Relationship between (A) BLA power (n = 95 sessions (11 mice)), (B) frontal cortex power (n = 89 sessions (11 mice)) with the amount of ethanol consumed in the DID-MSA binge periods. Download Figure 3-2, TIF file. [file eneuro-11-ENEURO.0017-24.2024-s004.tif]
